# The sensory-reactivity PROM set: identification of a parent reported outcome measure set for autism spectrum disorder

**DOI:** 10.1186/s41687-021-00396-w

**Published:** 2021-11-17

**Authors:** Dorinde M. van Andel, Henk F. van Stel, Floortje E. Scheepers, Kim J. Oostrom, Lotte Haverman, Hilgo Bruining

**Affiliations:** 1 Department of Psychiatry, UMC Utrecht Brain Center, University Medical Center Utrecht, Utrecht, The Netherlands; 2 Present Address: Department of Healthcare Innovation and Evaluation, Julius Center for Health Sciences and Primary Care, University Medical Center Utrecht, Utrecht, The Netherlands; 3 Child and Adolescent Psychiatry and Psychosocial Care, Emma Children’s Hospital, Amsterdam UMC, Amsterdam Reproduction and Development, Vrije Universiteit Amsterdam, Amsterdam, The Netherlands; 4 Child and Adolescent Psychiatry and Psychosocial Care, Emma Children’s Hospital, Amsterdam UMC, Vrije Universiteit Amsterdam, N=You centre, Levvel, Amsterdam Neuroscience, Amsterdam Reproduction and Development, Vrije Universiteit Amsterdam, Meibergdreef 9, 1105 AZ Amsterdam, The Netherlands; 5 Child and Adolescent Psychiatry and Psychosocial Care, Emma Children’s Hospital, Amsterdam UMC, Amsterdam Public Health, University of Amsterdam, Amsterdam, The Netherlands

**Keywords:** ASD, Sensory reactivity problems, PROM, PROMIS, Trials

## Abstract

**Background:**

Most children with autism spectrum disorder (ASD) suffer from aberrant responses to sensory stimuli that significantly impact the quality of life. To develop sensory interventions, individually tailored outcome measures are crucially needed for the domain of sensory reactivity problems. Here, we describe the identification of relevant sensory themes according to caregivers of children with ASD according to the guidelines for developing a (parent proxy) patient-reported outcome measure set. Subsequently, we identify parallels between these themes and a well-validated and supported PROMIS® portal to facilitate implementation. Interviews with clinicians and focus groups and interviews with parents of children with ASD were used in the initial phase for concept elicitation. Codes and themes were generated by qualitative thematic data analysis on the transcripts and cognitive interviews with different parents were used for revisions. The resulting themes were compared to existing generic PROMIS-item banks and other existing questionnaires.

**Results:**

A total of 11 parent-reported outcomes were identified that could be either classified as directly or indirectly related to sensory reactivity. Directly related themes comprised of: (1) sensory stimulation tolerance and (2) sensitivity to sensory stimuli. Indirectly related themes were: (3) irritable behavior (4) anxiety problems (5) mood problems (6) sleep problems (7) fatigue (8) physical complaints (9) daily functioning and participation (10) routines, structure and dealing with change and (11) problems in social interaction and communication. Seven out of 11 themes could be measured with generic PROMIS item banks. The four remaining outcomes (sensory stimulation tolerance; irritable behaviour; routines, structure and dealing with change; and sensitivity to sensory stimuli) were found suitable to be inventoried by existing PROMs.

**Conclusion:**

The majority of parent-reported problems seemed related to *indirect* consequences of sensory reactivity, which are suitable to be measured with generic item banks. In sum, we identified a sensory-reactivity PROM (parent-proxy) set consisting of PROMIS® item banks and additional domains that together form a comprehensive and readily available outcome set for sensory reactivity problems in children with ASD.

## Background

The majority of children with autism spectrum disorder (ASD) display aberrant responses to sensory experiences compared to their typically developing peers [1]. These responses are commonly referred to as sensory reactivity problems (SRPs) and are estimated to occur in 69–95% of patients with ASD [2, 3]. SRPs often hamper opportunities to participate in daily activities that promote learning [4, 5] and have a significant impact on quality of life for both children and caregivers [6, 7]. Intensive behavioral therapies such as the Early Start Denver Model [8] and Pivotal Response Treatment [9] partly rely on improving SRPs. In addition, novel mechanism-based medications are being developed to ameliorate SRPs through effects on neuronal activity [10–12]. Overall, interventions for SRPs are seen as a top priority in the ASD community [13].

To study the effect of existing and future interventions for SRPs, reliable and relevant outcome measures are mandatory, preferably from the patient or parent perspective to assess meaningful effects in daily life. Several existing instruments characterize sensory sensitivity behaviors such as the Sensory Profile [14] and the Evaluation of Sensory Processing [15] that have great utility for diagnostic profiling especially in typically developing populations. For repeated outcome measurements in clinical intervention trials, such in ASD, the questionnaires are rather lengthy and not well suited to detect change [16]. In addition, these questionnaires were mostly developed for typically developing populations and may be less suitable to address specific SRPs in clinical populations. Indeed, sensory reactivity problems in these populations can extend into problematic behavior or affective dysregulation that in turn may lead to some of the core symptoms of ASD [16]. In sum, there is a great need for appropriate outcome measures for treatments targeting SRPs in ASD.

To address this need, we set out to identify a comprehensive set of sensory reactivity related outcomes relevant to ASD. We first followed the steps of developing a (parent proxy) patient reported outcome measure (PROM) to be used as endpoint to establish efficacy in clinical trials [17, 18]. Our aim was to understand concepts that are relevant for patients and their caregivers. Our questions of interest therefore focused on what caregivers find relevant about sensory reactivity difficulties; which aspects have the most impact on their child’s and their own life; and how they would notice improvement when an intervention targeting sensory reactivity would be successful. To inventory these issues, we initiated focus groups and interviews with caregivers of children with ASD and sensory reactivity problems. We then assessed face validity of these concepts against currently existing instruments, which would facilitate their implementation in clinical trials. Therefore, we chose to compare our patient/caregiver relevant concepts to the items from the child and parent-proxy item banks from the *Patient-Reported Outcomes Measurement Information System®* (PROMIS) [19]. The PROMIS was initiated by the “NIH Roadmap Initiative” and has developed large item banks, based on Item Response Theory (IRT) with the possibility to use Computerized Adaptive Testing (CAT) [20] to develop meaningful, reliable and precise outcome measures which can be used internationally and across disorders. These properties enable easy and direct implementation of PROMIS CATs in clinical trials.

In this report we describe the identification of patient/caregiver relevant concepts and the development of a Sensory Reactivity-PROM set using PROMIS as a first milestone in establishing ecologically valid outcome measures for sensory reactivity.

## Methods

### Selection of PRO by concept elicitation

Ethical approval was granted by the Medical Ethical Committee of the University Medical Center (UMC) Utrecht. We selected parents/caregivers as respondents to be able to assess children from the age of 5 years of age. Parents of children with ASD were invited via advertisement on the Dutch ASD parent association (NVA) website to attend a focus group or interview over the phone. The inclusion criteria were parents of patients (boys and girls) with a confirmed ASD diagnosis (based on the DSM-IV or DSM-5) varying between 5 and 17 years of age. To obtain a heterogeneous and representative sample, no exclusion criterion based on intellectual functioning of patients and levels of parental education were selected. During the first steps of the qualitative phase, the aims and logistics of the PROM were developed to aid *concept elicitation*. Concept elicitation is a process by which concepts deemed important to patients and parents (i.e. symptoms as well as the impact of symptoms) emerge spontaneously through open-ended questions in interview settings, for instance in focus groups. First, symptoms of and impact of symptoms on children with ASD and their parents visiting the outpatient Psychiatry department for consultation or participating in scientific research at the ‘Care and Research program Sensory Processing’ between September 2016 and March 2019 (n = 200) were reviewed (case files). Second, structured expert brainstorms with 5 child psychiatrists from the department of Psychiatry of the UMC Utrecht were conducted to gather information from clinicians that worked with children with ASD. Lastly, a focus group (n = 8) and interviews over the phone (n = 10) with caregivers of children with ASD were conducted by following a structured interview guide. These conversations were transcribed and entered into Nvivo once the phone interviews reached data saturation (i.e. the point where no new themes or topics were obtained from further interviews).

A qualitative thematic data analysis [21], based on the method of Boeije [22] was performed on the transcription of phone interviews. A total of 181 codes were generated by DA and scored by an independent second rater (GT) and these were conceptually grouped into 29 (sub)themes. As a result, eleven overarching themes were extracted. Subsequently, these themes were presented to 21 new participants (i.e., other parents of children with ASD) to evaluate whether themes were missing or irrelevant. The eleven themes were confirmed and therefore maintained.

## Results

The study population that was interviewed and used for concept elicitation consisted of 38 caregivers of 37 children (age M = 11.5; SD = 3.0) with an ASD diagnosis. The population was balanced with regard to older and younger children (age 5–11: *n* = 19; age 12–17: *n* = 18) and boys (*n* = 19) and girls (*n* = 18). Intellectual functioning ranged from total intelligence quotient (TIQ) 50 to 145 (M = 98.9; SD = 28.2) and was proportionally divided into children with below average (TIQ 50–84: *n* = 10), average (TIQ 85–115: *n* = 10), and above average (TIQ 116–145: *n* = 8) intellectual functioning. Caregivers were most likely to have followed higher education: 56% completed higher professional education and 11% completed research-oriented education. Vocational education was completed by 30% and 4% had followed pre-vocational education.

The following eleven concepts were identified through concept elicitation: (1) Sensory stimulation tolerance (2) Sensitivity to sensory stimuli (3) Irritable behavior (4) Anxiety (5) Mood problems (6) Sleep problems (7) Fatigue (8) Physical complaints (9) Daily functioning and participation (10) Routines, structure and dealing with change and (11) Problems in social interaction and communication. Thus, the majority of these concepts reflected what we would refer to as ‘indirect’ consequences of altered sensory reactivity. For instance, many caregivers regarded their children’s fatigue and lack of energy to be closely related to aberrant sensory reactivity and to have a profound impact on the quality of life. Another example of indirect consequences were irritable behaviours, especially when their child experienced “sensory overload” leading to anger, temper tantrums, crying, yelling or short-temperedness. Only two themes—Sensory stimulation tolerance and Sensitivity to sensory stimuli—entailed direct behavioral responses to sensory stimuli, e.g., covering their ears, adjusting their daily routines or contacts to avoid sensory stimuli, or finding it difficult to differentiate between relevant and irrelevant stimuli.

### PROM selection

Next, we identified which reliable existing PROMs can be used to represent the identified sensory concepts. We chose to use PROMIS item banks, measured by CATs. Seven out of eleven concepts were found to be covered by PROMIS item banks (see Table 1). Importantly, each of these PROMIS item banks are available as parent proxies and pediatric self-report versions. Four concepts were not measurable by PROMIS: Sensory Stimulation Tolerance; Irritable Behaviour; Routines, Structure and Dealing with Change and Sensitivity to Sensory Stimuli. The domain Daily Functioning and Participation was only partly covered by the PROMIS items bank ‘Cognitive function’ and therefore an additional questionnaire would be needed to cover all codes that parents reported. The identification of relevant sensory reactivity PROs and PROMs resulted in a Sensory Reactivity-PROM set.

**Table 1:** Final sensory reactivity-PROM set

Relevant outcomes	PROMIS item banks	Validated PROM (sub) scales
*Directly related to sensory reactivity*		
1. Sensory stimulation tolerance		SSP or SEQ-3.0
2. Sensitivity to sensory stimuli		SSP or SEQ-3.0
*Indirectly related to sensory reactivity*		
3. Irritable behavior		ABC-Irritability
4. Anxiety	Anxiety (v2.0) Psychological stress experiences (v1.0)	
5. Mood problems	Depressive symptoms (v2.0) Life satisfaction (v1.0)	
6. Sleep problems	Sleep-related impairment (v1.0) Sleep disturbance (v1.0)	
7. Fatigue	Fatigue (v2.0)	
8. Physical complaints	Physical stress experiences (v1.0)	
9. Daily functioning and participation	Cognitive function (v.1.1)	CASP or PEM-CY
10. Routines, structure and dealing with change		RBS-R Ritualistic Behavior RBS-R Sameness Behavior
11. Problems in social interaction and communication	Peer relationships (v2.0) Family relationships (v1.0)	

ABC, Aberrant Behavior Checklist; CASP, Child and Adolescent Scale of Participation; PEM-CY, Participation and Environment Measure for Children and Youth; RBS-R, Repetitive Behavior Scale-Revised; SSP, Short Sensory Profile

### The sensory reactivity-PROM set

To measure Sensory stimulation tolerance and Sensitivity to sensory stimuli, we advise to use the following two questionnaires:

#### Short sensory profile (SSP)

The SSP is a shortened form of Dunn’s SP caregiver questionnaire [14] and contains 38 items, arranged into 7 subscales, aimed at measuring abnormal responses to sensory stimuli [23]. The normative group consists of 697 children from the United States. Both the SP full version as well as the SSP have shown good content validity [24]. It has a reliability of 0.90 and discriminate validity > 95% to identify children with and without sensory processing difficulties [25]. Although the total score is reliable for youth with ASD (α = 0.89), the structural validity of the SSP subscales shows poor fit [26, 27] and some researchers have recommended against the use of the SSP total score due to the measure’s multidimensionality [27]. The raw scores of both subscales and total scores are classified in three groups: typical performance, probable difference, and definite difference. A total score between 38 to 141 is classified as “definite difference” and indicates significant problems with processing sensory stimuli and difficulties in daily life performance which can be viewed as a clinically meaningful threshold.

#### Sensory Experiences Questionnaire Version 3.0 (SEQ-3.0)

Unlike other instruments that are often used in sensory processing research (e.g., SP-NL [14] and Sensory Processing measure [28], the SEQ-3.0 [29] was developed and standardized in ASD populations. Earlier versions of the SEQ have demonstrated its reliability and validity (version 1: Internal consistency α = 0.80; test–retest reliability total score ICC = 0.92 [30]). Studies about the content validity of the SEQ-3.0 have not been published although the developmental process is known (i.e., the items included were developed from reviews of ASD sensory literature and through a consensus process with a team of experts) [31]. Higher scores indicate greater symptoms, but no clinically meaningful threshold is established yet.

To measure Irritable behavior, we advise to use the following subscale:

#### Aberrant behavior checklist-irritability (ABC-I)

The ABC measures problematic behavior and contains 58 items organized into 5 subscales [32]. The ABC is developed as a scale to assess treatment effects in people with developmental disabilities (primarily in residential facilities) [32] and has very good internal consistency (α = ranging from low 0.80 s to the middle 0.90 s) and test–retest reliability (mid-0.60 s to highs in the 0.90 s) [33, 34]. Studies have further given psychometric support for the use of the ABC in ASD [35]. The Irritability subscale consists of 15 items and measures agitated/irritable behavior. This subscale has been a primary outcome to measure treatment response in large ASD trials [36–39] and psychosocial intervention studies [40, 41]. Higher scores indicate greater symptoms. The ABC does not have a clinically meaningful cut-off score, although pharmacological trials that included the Irritability subscale often required a baseline score of ≥ 18 [36, 42, 43].

To measure Routines, structure and dealing with change, we advise to use the following subscale:

#### Repetitive behavior scale-revised (RBS-R)

The RBS-R is a measure of the presence and severity of restricted and repetitive behaviors and contains 43 items organized into 6 subscales [44]. This refined version of the RBS was initiated after feedback from parents and clinicians and was developed to more appropriately capture the variety of repetitive behaviors in ASD. The subscales Ritualistic Behavior (i.e., performing activities of daily living in a similar manner) and Sameness Behavior (i.e., resistance to change, insisting things stay the same) have been selected. Internal consistency of these subscales in an ASD sample was α = 0.71 and 0.88, respectively [45]. Higher scores indicate more severe problems, but no clinically meaningful threshold has been established.

To measure Daily Functioning and Participation, the following two questionnaires can be used:

#### Child and adolescent scale of participation (CASP)

The CASP is a 20-items caregiver questionnaire measuring the extent of participation and restriction of children (3–22 years) in home, school and community life situations and activities [46]. The scale was initially developed in children with acquired brain injuries. The questionnaire has shown high internal consistency (Cronbach’s α = 0.98), test–retest reliability (ICC = 0.94) and construct validity [47, 48], and good to excellent content validity [49]. Lower scores indicate a lower extent of participation but a clinically meaningful threshold for ASD has not been established as a study on responsiveness is only available in children with traumatic brain injury [50]. A study with the German CASP version did report reference values for disability-free children, with recommended cut-off scores of < 95 for mildly and < 92 for severely impaired social participation [49].

#### Participation and environment measure for children and youth (PEM-CY)

The PEM-CY is a 25-items caregiver questionnaire that measures participation across life situations at home, school and community settings in children (5–17 years) with and without disabilities [51]. It has been developed in caregivers of both children with diverse disabilities (with a large proportion diagnosed with ASD) and without disabilities [52]. The questionnaire has both moderate to good internal consistency (Cronbach’s α = 0.59 and above) and test–retest reliability (0.58 and above) [52]. No data on clinically meaningful thresholds is available.

## Discussion

This study aimed to derive a valid outcome measure set for sensory reactivity targeting interventions in children with ASD elicited by parent interviews. The parent interviews and focus groups revealed a total of eleven concepts relating to SRPs. We can classify the most frequently mentioned problems as *indirect* consequences of sensory reactivity, such as fatigue or behavioral irritability. Interestingly, these kinds of symptoms are often recognized as comorbid features, but were here identified by parents as indirect consequences of altered sensory processing functions associated with ASD [16, 53]. In contrast, other behavioral responses were noted that were *directly* related to the sensory environment such as immediate distress or an exaggerated avoidance to sensory stimuli. In all, we classified nine out of 11 concepts as indirect versus two direct consequences of sensory reactivity. Such a distinction has not yet been explicated in the field [16, 53], but may be important to fully appreciate the effect of SRP targeting treatments.

The majority of the identified concepts (7 out of 11) can be measured with generic item banks provided by PROMIS. It would be recommended to validate these item banks in the target population (children with ASD). This set can be administered by CATs with both parent proxy reports (ages 5–17) and pediatric self-reports (ages 8–17) available. These CATs are an important method to reduce the time to complete questionnaires and to render them more individually tailored. CATs follow decision trees by each time choosing tailored selections based upon previous answers of the respondent, e.g. if a patient is unable to walk then all questions on mobility are deemed irrelevant. CAT algorithms hereby maximize the efficiency of number of questioned items (usually 4 to 12 items in PROMIS) and reduces the burden for respondents whilst allowing more domains to be measured. Another advantage of CAT in clinical trials is that smaller sample sizes are needed to achieve the same statistical power in comparison to conventional instruments. Lastly, there is less floor or ceiling effect with T-scores and different domains can be compared on the same scale. Thus, using PROMIS item banks for ASD is an important way forward since it is more reliable and reduces administration time for respondents and researchers. Hence, they are suitable to be implemented in clinical trials where multiple and repeated assessments are often desirable. In addition, using these generic item banks allow for comparisons between different (rare) disorders, typically developing children as well as comparisons across different countries.

A number of other concepts are not covered by existing PROMIS item banks at present: tolerance and sensitivity to sensory stimuli, behavioral irritability and problems with structure and dealing with change. To assemble an all-encompassing outcome measure set, these outcomes need to be added and (for the time-being) to be measured with other validated PROMs. To this end, we propose to add six additional, existing PROMs to cover the above-mentioned missing outcomes: the ABC-I subscale to assess irritable behavior, the SSP or SEQ-3.0 to assess items in the missing sensory-specific outcomes, the RBS-R Ritualistic Behavior and Sameness Behavior-subscales to address dealing with routine and change and the CASP or PEM-CY to cover the daily functioning and participation outcome. We do acknowledge that some of these measures have been developed more than 40 years ago and may not be in line with regulatory qualification demands in order to be implemented (immediately) in registrational clinical trials. Unfortunately, there are currently only few measures that have been validated and cover the identified relevant concepts. Indeed, a limitation of this study is the extensive variety and number of instruments that are needed to cover all relevant concepts identified in this study, which poses a significant burden on respondents. These results therefore highlight the need for the development of PRO instruments in the ASD population, that capture relevant concepts and have established clinically meaningful thresholds. Ultimately, the goal would be to fully rely on PROMs administered with CAT to comprise all identified concepts in the sensory reactivity domain.

## Conclusion

In conclusion, we bring forward a sensory reactivity-PROM set that addresses the main concepts relevant to parents of children with ASD. Through parent concept elicitation, we emphasize the need to measure both direct and indirect consequences of altered sensory reactivity. We hope that this report inspires ASD researchers to implement relevant outcome measures in their clinical trials with instruments that are user-friendly, less time-consuming and measure patient-relevant outcomes relating to sensory reactivity. In the short term, we suggest clinical trials in ASD focusing on SRPs to include this hybrid PROM set of PROMIS items banks and existing questionnaires. For the longer term, we propose to complete the PROM set by transforming conventional scales to reliable PROMs administered by CATs.

## Data Availability

The transcripts generated and analyzed during the current study are not publicly available due to privacy reasons of participants but are available from the corresponding author on reasonable request.

## Abbreviations

ASD: Autism spectrum disorder
CAT: Computerized adaptive testing
DSM-5: Diagnostic and statistical manual of mental disorders 5th edition
IRT: Item response theory
PROM: Patient-reported outcome measure
PROMIS: Patient-reported outcomes measurement information system
SRPs: Sensory reactivity problems
